# Author Correction: Structural basis of the histone ubiquitination read–write mechanism of RYBP–PRC1

**DOI:** 10.1038/s41594-025-01674-7

**Published:** 2025-08-27

**Authors:** Maria Ciapponi, Elena Karlukova, Sven Schkölziger, Christian Benda, Jürg Müller

**Affiliations:** 1 Laboratory of Chromatin Biology, Max-Planck Institute of Biochemistry, Martinsried, Germany; 2 Department of Structural Cell Biology, Max-Planck Institute of Biochemistry, Martinsried, Germany

**Keywords:** Cryoelectron microscopy, Histone post-translational modifications, Chromatin, Ubiquitylation, Ubiquitylation

Correction to: *Nature Structural & Molecular Biology* 10.1038/s41594-024-01258-x, published online 25 March 2024.

After publication, the authors noticed that in Fig. 1h and once in the text (in the third paragraph, “The resulting model shows RYBP NZF residues T31, F32 and I25…”), they had mistakenly labeled an RYBP isoleucine in the RYBP-Ubiquitin interface as I25. The correct number is I43. This was an inadvertent labeling error and does not impact any of the conclusions. The figure and text have now been corrected in the HTML and PDF versions of the article. The authors apologize for this mistake and any confusion that it may have created.

