# Supplementary material for: Structural basis of the histone ubiquitination read–write mechanism of RYBP–PRC1
Source: Nat Struct Mol Biol. 2024 Mar 25;31(7):1023–7. doi: 10.1038/s41594-024-01258-x (PMC11257959; doi:10.1038/s41594-024-01258-x)
Supplement: Supplementary file 1 — Reporting Summary [file 41594_2024_1258_MOESM1_ESM.pdf]

nature portfolio

Corresponding author(s): Jürg Müller
Christian Benda

Last updated by author(s): Feb 1, 2024

Reporting Summary

Nature Portfolio wishes to improve the reproducibility of the work that we publish. This form provides structure for consistency and transparency in reporting. For further information on Nature Portfolio policies, see our Editorial Policies and the Editorial Policy Checklist.

Statistics

For all statistical analyses, confirm that the following items are present in the figure legend, table legend, main text, or Methods section.

n/a	Confirmed	
☐	☒	The exact sample size (*n*) for each experimental group/condition, given as a discrete number and unit of measurement
☐	☒	A statement on whether measurements were taken from distinct samples or whether the same sample was measured repeatedly
☒	☐	The statistical test(s) used AND whether they are one- or two-sided<br>*Only common tests should be described solely by name; describe more complex techniques in the Methods section.*
☒	☐	A description of all covariates tested
☒	☐	A description of any assumptions or corrections, such as tests of normality and adjustment for multiple comparisons
☐	☒	A full description of the statistical parameters including central tendency (e.g. means) or other basic estimates (e.g. regression coefficient) AND variation (e.g. standard deviation) or associated estimates of uncertainty (e.g. confidence intervals)
☒	☐	For null hypothesis testing, the test statistic (e.g. *F*, *t*, *r*) with confidence intervals, effect sizes, degrees of freedom and *P* value noted<br>*Give P values as exact values whenever suitable.*
☒	☐	For Bayesian analysis, information on the choice of priors and Markov chain Monte Carlo settings
☒	☐	For hierarchical and complex designs, identification of the appropriate level for tests and full reporting of outcomes
☒	☐	Estimates of effect sizes (e.g. Cohen's *d*, Pearson's *r*), indicating how they were calculated

Our web collection on statistics for biologists contains articles on many of the points above.

Software and code

Policy information about availability of computer code

Data collection	SerialEM 4.0
Data analysis	RELION 3.1<br>MotionCorr2 1.1<br>CTFFIND 4.1.13.<br>Coot 0.9.8.7<br>PHENIX 1.20<br>UCSF ChimeraX 1.4.<br>ImageJ 12.24.12<br>Matlab R2022B<br>Prism9 9.5.1<br>DeepEMhancer<br>Image Lab v6.1

For manuscripts utilizing custom algorithms or software that are central to the research but not yet described in published literature, software must be made available to editors and reviewers. We strongly encourage code deposition in a community repository (e.g. GitHub). See the Nature Portfolio guidelines for submitting code & software for further information.

Data

Policy information about availability of data

All manuscripts must include a data availability statement. This statement should provide the following information, where applicable:

- Accession codes, unique identifiers, or web links for publicly available datasets
- A description of any restrictions on data availability
- For clinical datasets or third party data, please ensure that the statement adheres to our policy

The protein structure data reported in this study have been deposited in PDB under the accession codes PDB ID 8PP6 and 8PP7 and in the EMDB under accession codes EMD-17796 and EMD-17797.
Initial models used for CryoEM model building (PDB code): 6PWE, 4R8P, 1Q5W

Research involving human participants, their data, or biological material

Policy information about studies with human participants or human data. See also policy information about sex, gender (identity/presentation), and sexual orientation and race, ethnicity and racism.

Reporting on sex and gender	N/A
Reporting on race, ethnicity, or other socially relevant groupings	N/A
Population characteristics	N/A
Recruitment	N/A
Ethics oversight	N/A

Note that full information on the approval of the study protocol must also be provided in the manuscript.

Field-specific reporting

Please select the one below that is the best fit for your research. If you are not sure, read the appropriate sections before making your selection.

☒ Life sciences ☐ Behavioural & social sciences ☐ Ecological, evolutionary & environmental sciences

For a reference copy of the document with all sections, see nature.com/documents/nr-reporting-summary-flat.pdf

Life sciences study design

All studies must disclose on these points even when the disclosure is negative.

Sample size	The numbers of particles used for cryo-EM reconstructions are indicated in Extended Data Table 1<br>For all EMSAs and enzyme activity assays, we conducted experiments with three or more replicates to ensure reproducibility.<br>The consistent results across all replicates suggests that the number of replicates was sufficient in all experiments.<br>No statistical methods were used to predetermine the sample size.
Data exclusions	No data were excluded
Replication	For quantitative measurements, experiments were performed at least in triplicate. All of them were successful.
Randomization	not relevant, no grouped samples
Blinding	not relevant, no grouped samples

Reporting for specific materials, systems and methods

We require information from authors about some types of materials, experimental systems and methods used in many studies. Here, indicate whether each material, system or method listed is relevant to your study. If you are not sure if a list item applies to your research, read the appropriate section before selecting a response.

Materials & experimental systems

n/a	Involved in the study
☒	☐ Antibodies
☐	☒ Eukaryotic cell lines
☒	☐ Palaeontology and archaeology
☒	☐ Animals and other organisms
☒	☐ Clinical data
☒	☐ Dual use research of concern
☒	☐ Plants

Methods

n/a	Involved in the study
☒	☐ ChIP-seq
☒	☐ Flow cytometry
☒	☐ MRI-based neuroimaging

Eukaryotic cell lines

Policy information about cell lines and Sex and Gender in Research

Cell line source(s)

Cell line (Trichoplusia ni)
HighFive cell line for expression Invitrogen Product nr.:
B85502
BTI-Tn-5B1-4
(RRID:CVCL_C190)
Protein expression
Cell line (Spodoptera frugiperda)
Sf21 cell line for
Baculovirus production
Invitrogen Product nr.:
1149701
(RRID:CVCL_0518)
Baculovirus production for protein expression

Authentication

Commercial cell lines. Authentication is performed by the manufacturer

Mycoplasma contamination

The cell line were not tested for mycoplasma contamination

Commonly misidentified lines
(See ICLAC register)

No commonly misidentified lines were used

Plants

Seed stocks

Report on the source of all seed stocks or other plant material used. If applicable, state the seed stock centre and catalogue number. If plant specimens were collected from the field, describe the collection location, date and sampling procedures.

Novel plant genotypes

Describe the methods by which all novel plant genotypes were produced. This includes those generated by transgenic approaches, gene editing, chemical/radiation-based mutagenesis and hybridization. For transgenic lines, describe the transformation method, the number of independent lines analyzed and the generation upon which experiments were performed. For gene-edited lines, describe the editor used, the endogenous sequence targeted for editing, the targeting guide RNA sequence (if applicable) and how the editor was applied.

Authentication

Describe any authentication procedures for each seed stock used or novel genotype generated. Describe any experiments used to assess the effect of a mutation and, where applicable, how potential secondary effects (e.g. second site T-DNA insertions, mosiacism, off-target gene editing) were examined.
