# Supplementary figures and images for: Structural basis of the histone ubiquitination read–write mechanism of RYBP–PRC1
Source: Nat Struct Mol Biol. 2024 Mar 25;31(7):1023–7. doi: 10.1038/s41594-024-01258-x (PMC11257959; doi:10.1038/s41594-024-01258-x)

Panel c:

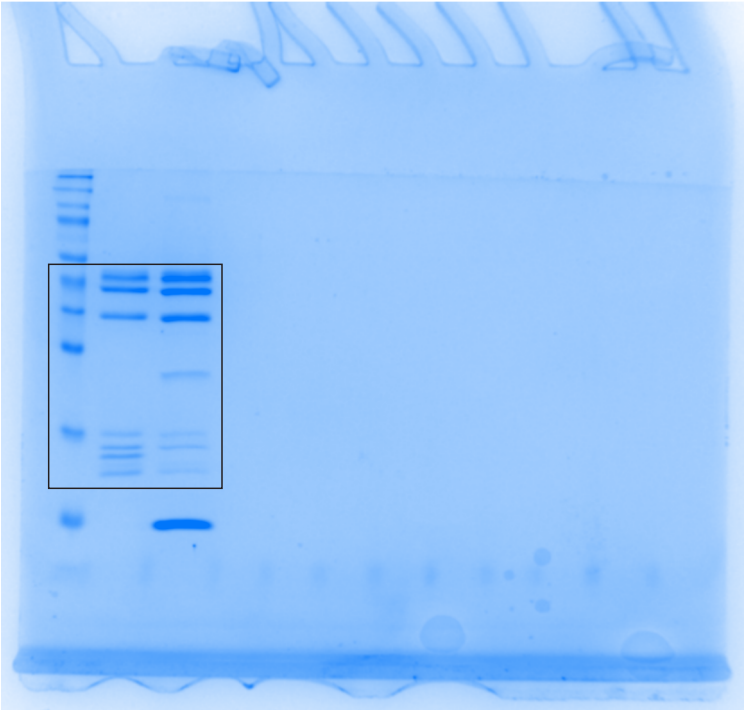

Supplement: Supplementary file 3 — Uncropped gel image. [file 41594_2024_1258_MOESM3_ESM.pdf]

Panel a:

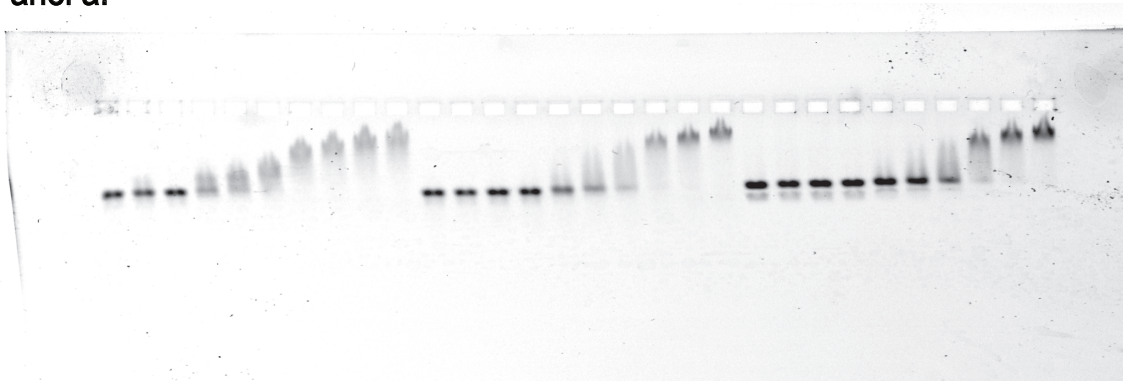

Panel b:

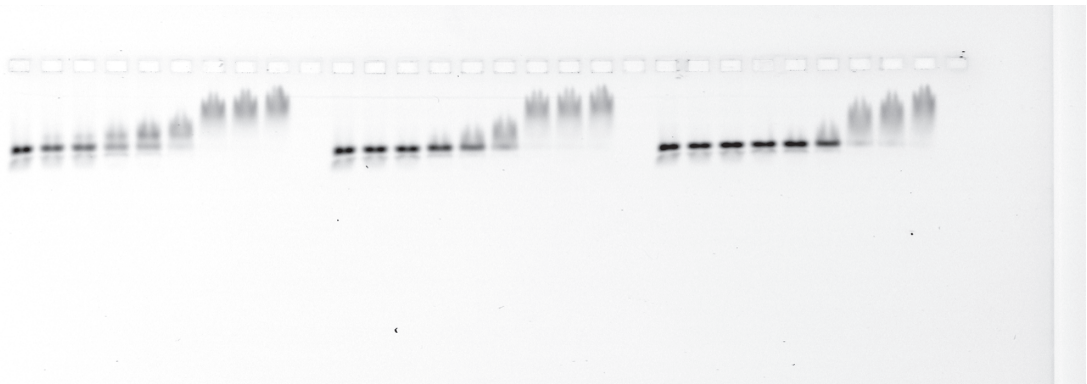

Panel c:

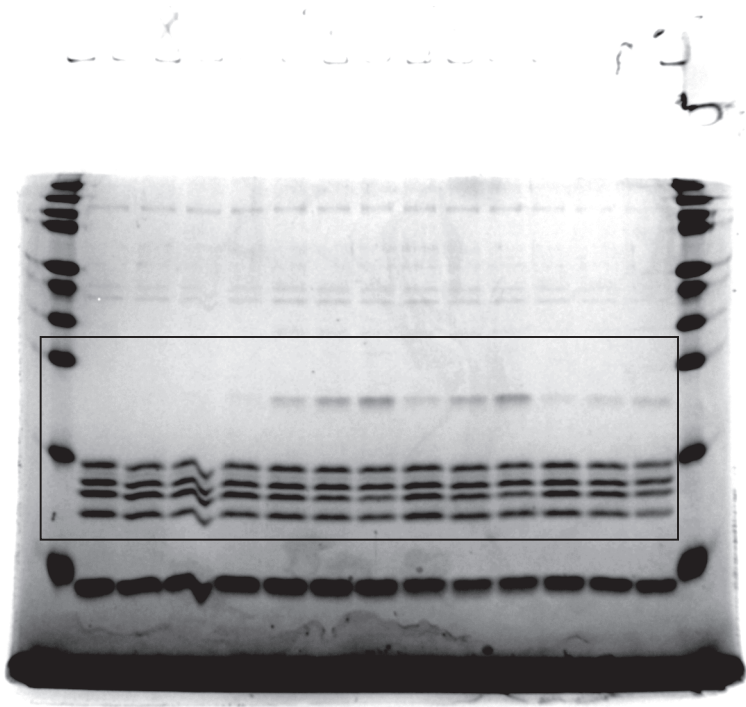

Supplement: Supplementary file 4 — Uncropped gel images. [file 41594_2024_1258_MOESM4_ESM.pdf]

Panel a:

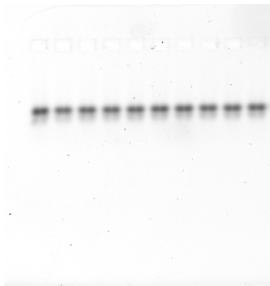

Panel b:

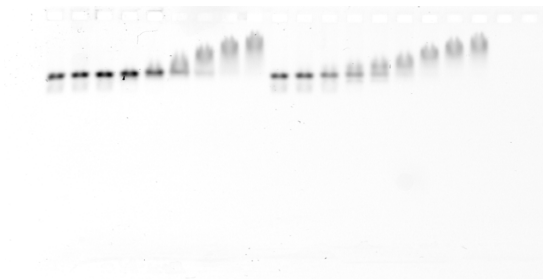

Panel c:

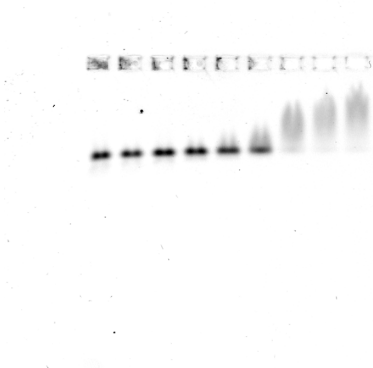

Supplement: Supplementary file 6 — Uncropped gel images. [file 41594_2024_1258_MOESM6_ESM.pdf]

Panel b:

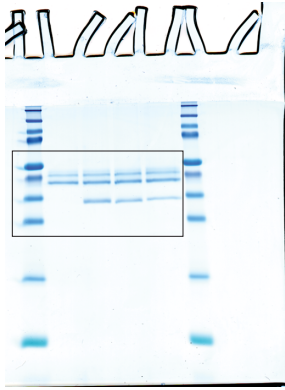

Panel c:

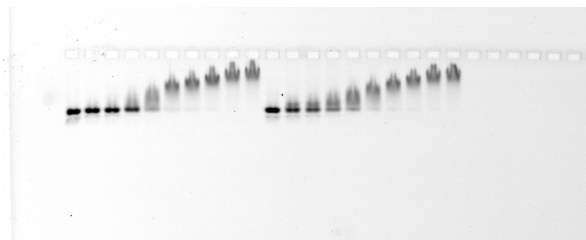

Panel d:

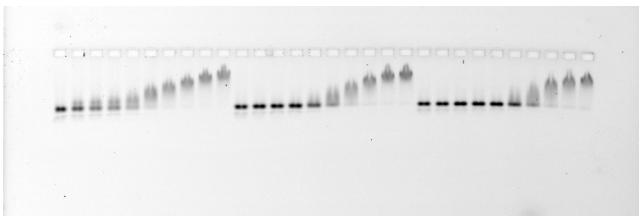

Supplement: Supplementary file 8 — Uncropped gel images. [file 41594_2024_1258_MOESM8_ESM.pdf]
